# Prescribing patterns in dementia: a multicentre observational study in a German network of CAM physicians

**DOI:** 10.1186/1471-2377-11-99

**Published:** 2011-08-08

**Authors:** Elke Jeschke, Thomas Ostermann, Horst C Vollmar, Manuela Tabali, Friedemann Schad, Harald Matthes

**Affiliations:** 1 Havelhoehe Research Institute, Kladower Damm 221, 14089 Berlin, Germany; 2 Center for Integrative Medicine, University of Witten/Herdecke, Gerhard-Kienle-Weg 4, 58313 Herdecke, Germany; 3 German Center for Neurodegenerative Diseases (DZNE), Stockumer Str. 12, 58453 Witten, Germany; 4 Institute for General Practice and Family Medicine, University of Witten/Herdecke, Alfred-Herrhausen-Str. 50, 58448 Witten, Germany

## Abstract

**Background:**

Dementia is a major and increasing health problem worldwide. This study aims to investigate dementia treatment strategies among physicians specialised in complementary and alternative medicine (CAM) by analysing prescribing patterns and comparing them to current treatment guidelines in Germany.

**Methods:**

Twenty-two primary care physicians in Germany participated in this prospective, multicentre observational study. Prescriptions and diagnoses were reported for each consecutive patient. Data were included if patients had at least one diagnosis of dementia according to the 10th revision of the International Classification of Diseases during the study period. Multiple logistic regression was used to determine factors associated with a prescription of any anti-dementia drug including *Ginkgo biloba*.

**Results:**

During the 5-year study period (2004-2008), 577 patients with dementia were included (median age: 81 years (IQR: 74-87); 69% female). Dementia was classified as unspecified dementia (57.2%), vascular dementia (25.1%), dementia in Alzheimer's disease (10.4%), and dementia in Parkinson's disease (7.3%). The prevalence of anti-dementia drugs was 25.6%. The phytopharmaceutical *Ginkgo biloba *was the most frequently prescribed anti-dementia drug overall (67.6% of all) followed by cholinesterase inhibitors (17.6%). The adjusted odds ratio (AOR) for receiving any anti-dementia drug was greater than 1 for neurologists (AOR = 2.34; CI: 1.59-3.47), the diagnosis of Alzheimer's disease (AOR = 3.28; CI: 1.96-5.50), neuroleptic therapy (AOR = 1.87; CI: 1.22-2.88), co-morbidities hypertension (AOR = 2.03; CI: 1.41-2.90), and heart failure (AOR = 4.85; CI: 3.42-6.88). The chance for a prescription of any anti-dementia drug decreased with the diagnosis of vascular dementia (AOR = 0.64; CI: 0.43-0.95) and diabetes mellitus (AOR = 0.55; CI: 0.36-0.86). The prescription of *Ginkgo biloba *was associated with sex (female: AOR = 0.41; CI: 0.19-0.89), patient age (AOR = 1.06; CI: 1.02-1.10), treatment by a neurologist (AOR = 0.09; CI: 0.03-0.23), and the diagnosis of Alzheimer's disease (AOR = 0.07; CI: 0.04-0.16).

**Conclusions:**

This study provides a comprehensive analysis of everyday practice for treatment of dementia in primary care in physicians with a focus on CAM. The prescribing frequency for anti-dementia drugs is equivalent to those found in other German studies, while the administration of *Ginkgo biloba *is significantly higher.

## Background

The management of dementia is a key challenge in modern health systems. The prevalence of dementia within people aged 65 years or older is 6.8% [1,2]. It increases with patient age and doubles every 5 years [3]. As the proportion of people aged 65 years or older rises from 16.8% in 2010 to 23.5% in 2050, the number of dementia cases will also increase from 1.21 million people today to 2.62 million people in 2050 in Germany alone [2]. It is estimated that the number of sufferers from dementia worldwide will increase from 35.6 million today to 65.7 million by 2030 and 115.4 million by 2050 [3]. The raise in dementia patients is directly correlated with increasing costs in the health care systems. While 7.1 million euros were expended in Germany for treatment and care of dementia in 2002, the costs increased to 7.8 million euros in 2004, 8.6 million euros in 2006, and finally 9.4 million euros in 2008 [4].

Alzheimer's disease and vascular dementia are the two most common types of age-related dementia. Alzheimer's disease accounts for more than 65% of dementia in the elderly [1]. At present no curative treatment is available. Interventions focus on slowing the course of the disease. Compared with placebo, pharmacological treatment with cholinesterase inhibitors and memantine has shown improvements in outcomes such as cognition and global functioning in Alzheimer's disease [5,6]. However, the clinical relevance of these treatment effects seems marginal [7,8].

Some studies have indicated that complementary and alternative medicine (CAM) from the areas of phytotherapy, traditional Chinese medicine, or homoeopathy has potential in the treatment of dementia [9-11]. In particular the phytopharmaceutical *Ginkgo biloba *is traditionally used quite frequently [12,13]. In Germany, in contrast to some other countries, *Ginkgo biloba *is available as prescription drug for the therapy of mild to moderate dementia. Although there is acceptable physiological compatibility and sufficiently proven safety, there is still an unclear picture of evidence [14,15]. According to the meta-analysis of Weinmann et al. the change of scores for cognition were in favour of *Ginkgo biloba *compared to placebo, but they did not show a statistically significant difference from placebo for activities in daily living [16]. The German Institute for Quality and Efficiency in Health Care (IQWIG) found only a small benefit for the 240 mg preparation and criticised the heterogeneity of the study results [17]. In fact, *Ginkgo biloba *is not yet included in evidence-based dementia guidelines published in Germany.

The development, dissemination, and implementation of these dementia guidelines are a key strategy for improving the care of persons with dementia. In Germany one guideline from the German Society of General Physicians (DEGAM) with a focus on family medicine was published in 2008 while another one from the German Society of Neurology and German Society of Gerontopsychiatry (DGN and DGPPN) mainly for specialists was finalised in 2009 [18,19]. Unfortunately both guidelines reveal different recommendations for the treatment of dementia, which might be due to the abovementioned inconsistent appraisal of clinical relevance from study results. The DEGAM guideline is much more cautious in the recommendation of cholinesterase inhibitors than the guideline of the DGN/DGPPN, and while memantine is recommended for Alzheimer's disease in the guideline of the DGN/DGPPN, the DEGAM only recommends it as an individual approach for therapy [20].

In therapy for vascular dementia, recommended therapies focus on the treatment of cardio-vascular risk factors, e.g. the prevention of prospective strokes. The treatment of vascular dementia with anti-dementia drugs, until now, did not produce a reliable portfolio of evidence and thus the DEGAM does not give any recommendations where the DGN/DGPPN allows for a selective therapeutic attempt in single cases. However, with respect to the high amount of elderly patients, guidelines mainly discuss the problems occurring with age-related co-morbidity and poly-pharmacy.

Apart from two concurrent German guidelines, an additional barrier to guideline adherence is the difficulty faced by physicians in reconciling patient preferences with guideline recommendations [21]. According to studies in a variety of primary care settings, patient expectations and preferences can influence the health care provided to them [22-24]. This issue may be particularly salient in the setting of CAM, as patients seeking treatment from physicians specialised in this field are likely to expect to receive some form of alternative therapy. Furthermore, one should not underestimate the impact of the physician's conviction on the prescription practice. In a survey of German GPs, ginkgo preparations for dementia were voted to be equally as effective as cholinesterase inhibitors and memantine [25].

The present study thus aims (a) to analyse prescribing patterns among primary care physicians specialised in CAM, b) to investigate conformity and variations in prescriptions according to the German dementia guidelines (DEGAM and DGN/DGNPP), c) to identify different treatment strategies in GPs and neurologists and d) provide information of co-morbidities of patients with dementia visiting primary health care physicians specialised in CAM.

## Methods

In total, 22 primary care physicians in Germany participated in this prospective, multicentre observational study. All of them were members of the EvaMed Network, which aims to evaluate CAM remedies in usual care with regard to prescribing patterns, efficacy, and safety [26-28]. Physicians were recruited through the German National Association of Anthroposophic Physicians (*Gesellschaft Anthroposophischer Ärzte in Deutschland*; GAÄD). A total of 362 physicians were contacted and informed about the EvaMed Network by standard mail and, in the event of non-response, 4 weeks later by telephone. For a physician to be eligible to participate in the study, his or her medical practice had to meet a number of technical requirements, including the presence of a special computerised patient documentation system (DocExpert, DocConcept, TurboMed, Duria, PDE-Top, Medistar), a local area network (LAN) connection, and Microsoft Windows and Internet Explorer (i.e. as client software). A total of 38 physicians (10.5%) fulfilled the technical requirements, gave informed consent, and agreed to participate in the EvaMed Network. Of these physicians, 16 specialised in paediatrics, dermatology, and gynaecology and were excluded from the study. Each of the remaining 22 physicians had practised for at least 5 years in addition to completing training in anthroposophic medicine.

The present study is based on secondary data provided by physicians for health insurance accounting. As such, the recommendations for good practice in secondary data analysis (e.g. anonymisation of data on prescriptions and diagnoses) developed by the German Working Group on the Collection and Use of Secondary Data [29] were applied in full.

**Figure 1:**
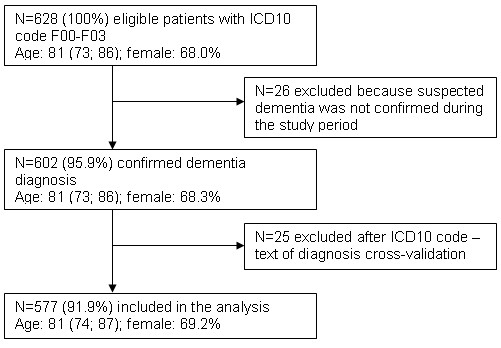
**Flow chart of the inclusion process**.

Data were included if patients had at least one diagnosis of dementia according to the 10th revision of the International Classification of Diseases (ICD10: F00-F03) during the 5-year study period (01.01.2004-01.01.2009). Data were excluded if the diagnosis dementia was only a suspected diagnosis and not confirmed during the study period and if a cross-validation of the ICD10 code and text of diagnosis indicated an incorrect coding. Figure 1 shows the flow chart of the inclusion process.

During the study, physicians continued to follow their routine documentation procedures, recording diagnoses and all prescriptions for each consecutive patient using their existing, computerised patient documentation system. These data were exported to the QuaDoSta postgreSQL database hosted in each practice [30]. Physicians used a browser-based interface to match individual diagnoses with the corresponding drugs or remedies that had been prescribed. Prescribed drugs were documented using the German National Drug Code. Diagnoses were coded according to the 10th revision of the International Classification of Diseases (ICD-10).

Dementia was classified as 'Dementia in Alzheimer's disease', 'Vascular dementia', 'Dementia in Parkinson's disease', and 'Unspecified dementia'. Co-morbidities were classified as hypertension, depression, heart failure, chronic ischemic heart disease, cardiac arrhythmias, diabetes mellitus, dyslipidemia, atherosclerosis, other cerebrovascular diseases, pressure ulcer, stroke, cerebral infarction, arthropathies, and dorsopathies. Multi-morbidity was considered if a patient had at least 2 co-morbidities.

Study investigators identified all drugs and remedies prescribed for dementia. Each substance was classified using the Anatomical Therapeutic Chemical Index (ATC) and anti-dementia drugs were clustered into cholinesterase inhibitors (i.e. donepezil, rivastigmine, galantamine), memantine, *Ginkgo biloba*, and older anti-dementia drugs (e.g. piracetam, nimodipine, and selegiline). Other medication was classified as antidepressants, anitpsychotics, benzodiazepine, anti-Parkinson drugs, antiepileptics, and sedatives.

Statistical analysis was performed with SPSS 18.0 for Windows. Descriptive analysis was used to determine prescription rates. Means and standard deviations (SD) were calculated for continuous, normal data. In cases where data were not normally distributed, medians and interquartile ranges (IQR) were reported. Subgroup analyses of prescribing rates were performed for patient age (under 60 years, 60-69 years, 70-79 years, 80-89 years, 90-99 years, 100 years and older), gender, and co-morbidities. The two-tailed Chi square test was used to analyse differences in prescription rates. A *P *value of less than 0.05 was regarded as indicating a statistically significant difference.

Adjusted odds ratios (AOR) and 95% confidence intervals (CI) were calculated using multiple logistic regressions to determine factors associated with a) a prescription of any anti-dementia drug and b) a prescription of *Ginkgo biloba *versus cholinesterase inhibitors and/or memantine. Age, sex, physician specialization (neurologist vs. GP), year of prescription, type of consultation (first vs. follow up), type of dementia, multi-morbidity, co-morbidities, neuroleptic, and antidepressant therapy were included as independent variables. Patient age was introduced in the model as a continuous variable. For the "best-fitting" logistic regression model, we employed the stepwise Akaike Information Criterion (AIC).

## Results

### Physicians

Of the 22 physicians, 17 were GPs (77%) and 5 were specialist GPs (23%). The participating physicians did not differ significantly from the overall population of physicians certified in anthroposophy in Germany (n = 362) in terms of age (mean = 49.4; SD = 6.3 years vs. mean = 47.5; SD = 6.1 years; *P *= 0.709) or gender (60.0% vs. 62.2% men; *P *= 0.917), and were only slightly younger and consisted of a similar percentage of women compared to all office-based physicians in Germany (mean 52.0 years; 61.2% men) [31].

### Study population

During the 5-year study period, a total of 577 patients with dementia were included. The inclusion process is shown in Figure 1. 374 patients (64.8% of all patients) were treated by a GP and the remaining 203 patients were treated by a neurologist. 69.2% of the patients were female. The median age of the patients was 81 years (IQR: 74, 87). Altogether, 4.9% of the patients were under 60 years (n = 28), 10.1% were 60-69 years (n = 58), 27.2% were 70-79 years (n = 157), 41.4% were 80-89 years (n = 239), 16.1% were 90-99 years (n = 93), and 0.3% were 100 years or older (n = 2). Dementia was classified as unspecified dementia (57.2%), vascular dementia (25.1%), dementia in Alzheimer's disease (10.4%), and dementia in Parkinson's disease (7.3%). Table 1 shows the sample of patients according to age, sex, and type of dementia.

**Table 1:** Patients according to age, sex, and type of dementia

Dementia	Patients	Age [years]	Sex [%]	
		
			Male	Female
	**N**	**Median (IQR)**	**%**	**%**

**Dementia in Alzheimer's disease**	**60**	79 (74.0; 85.0)	35.0	65.0

**Vascular dementia**	**145**	77 (69.5; 83.5)	33.1	66.9

**Dementia in Parkinson's disease**	**42**	79 (74.5; 84.3)	38.1	61.9

**Unspecified dementia**	**330**	83 (76.5; 88.0)	28.1	71.8

**Total**	**577**	**81 (74.0, 87.0)**	**30.8**	**69.2**

In total, 76.4% of all patients (n = 441) had two or more co-morbidities and were therefore classified as multi-morbid. The most frequent co-morbidities were hypertensive diseases (43.5% of all patients), other forms of heart disease (38.8%), dorsopathies (30.3%), arthropathies (29.8%), mood [affective] disorders (27.7%), and ischemic heart diseases (23.1%). Table 2 provides a detailed overview of the most frequent co-morbidities of the participating patients according to type of dementia.

**Table 2:** Sample of patients with dementia subdivided according to dementia-relevant co-morbidities

Dementia	Patients	Co-morbidities ^1 ^										
		
		Hyper-tension	Depression	Heart failure	Chronic ischaemic heart disease	Diabetes mellitus	Dys-lipidemia	Cardiac arrhythmias	Athero-sclerosis	Stroke or cerebral infarction	Other cerebrovascular diseases ^2^	Pressure ulcer
	N	[n (%)]	[n (%)]	[n (%)]	[n (%)]	[n (%)]	[n (%)]	[n (%)]	[n (%)]	[n (%)]	[n (%)]	[n (%)]
**Dementia in Alzheimer's disease**	**60**	24 (40.0)	20 (33.3)	21 (35.0)	15 (25.0)	9 (15.0)	16 (26.7)	11 (18.3)	12 (20.0)	8 (13.3)	11 (18.3)	9 (15.0)

**Vascular dementia**	**145**	66 (45.5)	40 (27.6)	23 (15.9)	38 (26.2)	30 (20.4)	28 (19.3)	25 (17.2)	34 (23.4)	29 (20.0)	22 (15.2)	6 (4.1)

**Dementia in Parkinson's disease**	**42**	14 (33.3)	17 (40.5)	13 (31.0)	4 (9.5)	8 (19.0)	5 (11.9)	6 (14.3)	6 (14.3)	11 (26.2)	7 (16.7)	8 (19.0)

**Unspecified dementia**	**330**	144 (43.6)	80 (24.2)	96 (29.1)	56 (17.0)	56 (17.0)	49 (14.8)	52 (15.8)	36 (10.9)	39 (11.8)	35 (10.6)	30 (9.1)

**Total**	**577**	**248 (43.0)**	**157 (27.2)**	**153 (26.5)**	**113 (19.6)**	**103 (17.9)**	**98 (17.0)**	**94 (16.3)**	**88 (15.3)**	**87 (15.1)**	**75 (13.0)**	**53 (9.2)**

^1 ^Double entries possible, sorted by descending frequency; ^2 ^including cerebral aneurysm (nonruptured, cerebral atherosclerosis, hypertensive encephalopathy, nonpyogenic thrombosis of intracranial venous system, and cerebral arteritis, not elsewhere classified

### Anti-dementia drug therapy

In total, 25.6% of all patients (n = 148) were prescribed anti-dementia drugs (i.e. cholinesterase inhibitors, memantine, or *Ginkgo biloba)*. Table 3 gives a detailed overview of the included patients according to anti-dementia drugs and type of dementia. Most of the patients received *Ginkgo biloba *(n = 99). 11.3% of all patients (n = 65) received an anti-dementia drug therapy with cholinesterase inhibitors or memantine. Cholinesterase inhibitors were prescribed for 40 patients. Donepezil was the most frequently prescribed cholinesterase inhibitor (n = 29), followed by galantamine (n = 10) and rivastigmine (n = 8). In total, 32 patients were prescribed memantine. Thirty patients were prescribed 2 or 3 different anti-dementia drugs, mostly in combination with *Ginkgo biloba *(memantine and *Ginkgo biloba *n = 10; cholinesterase inhibitors and *Ginkgo biloba *n = 9; cholinesterase inhibitors and memantine n = 8, cholinesterase inhibitors, memantine and *Ginkgo biloba *n = 4). Only 6 patients received older anti-dementia drugs like piracetam, nimodipine, and selegiline.

**Table 3:** Sample of patients with dementia subdivided according to anti-dementia drugs

Dementia	Patients	Anti-dementia drugs ^1^					
		
		Total	Cholinesterase inhibitors			Memantine	Ginkgo biloba
		
			Donepezil	Rivastigmine	Galantamine		
	**N**	**[n (%)]**	**[n (%)]**	**[n (%)]**	**[n (%)]**	**[n (%)]**	**[n (%)]**

**Dementia in Alzheimer's disease**	**60**	34 (56.7)	13 (21.7)	3 (5.0)	7 (11.7)	15 (25.0)	12 (20.0)

**Vascular dementia**	**145**	26 (17.9)	1 (0.7)	-	1 (0.7)	1 (0.7)	23 (15.9)

**Dementia in Parkinson's disease**	**42**	8 (19.0)	1 (2.4)	1 (2.4)	1 (2.4)	2 (4.8)	5 (11.9)

**Unspecified dementia**	**330**	80 (24.2)	14 (4.2)	4 (1.2)	1 (0.3)	14 (4.2)	59 (17.9)

**Total**	**577**	**148 (25.6)**	**29 (5.0)**	**8 (1.4)**	**10 (1.7)**	**32 (5.5)**	**99 (17.2)**

^1 ^Double entries possible

56.7% of patients with dementia with Alzheimer's disease received anti-dementia drugs, whereas less than 25% of patients with other dementia types were prescribed anti-dementia drugs (Table 3). Donepezil and memantine were prescribed mostly for patients with dementia with Alzheimer's disease. The phytotherapeutical *Ginkgo biloba *was prescribed for patients with dementia with Alzheimer's disease, vascular dementia, and unspecified dementia in equal shares (16% - 20%). Patients with vascular dementia were prescribed *Ginkgo biloba *(15.9% *Ginkgo biloba *vs. 2.0% of any other anti-dementia drug) nearly exclusively.

In total, 448 prescriptions of anti-dementia drugs for the 148 patients who received at least one of these drugs were reported. The phytopharmaceutical *Ginkgo biloba *was the most frequently prescribed anti-dementia drug overall (67.6% of all anti-dementia drugs).

### Prescriptions for dementia-related co-morbidities

The most frequent medication prescribed to patients for dementia-relevant co-morbidities were antidepressants (20% of all patients), antipsychotics (15%; thereof risperidone 7%, butyrophenone 7%), and benzodiazepine (10%). Table 4 provides a detailed overview of these medications according to type of dementia. In total, 18 patients received the antidepressant CAM remedy *Hypericum perforatum *(14.6% of all patients with antidepressants). The antipsychotic risperidone was prescribed for 7 patients with dementia with Alzheimer's disease, 7 patients with vascular dementia, 2 patients with dementia with Parkinsons's disease, and 24 patients with unspecified dementia. Of the 40 patients being prescribed cholinesterase inhibitors, only one received additional anticholinergic agents during the study period.

**Table 4:** Sample of patients with dementia subdivided according to medication prescribed for dementia relevant co-morbidities

Dementia	Patients	Medication ^1 ^					
		
		Antidepressants	Antipsychotics	Benzodiazepine	Anti-Parkinson drugs	Antiepileptics	Sedatives
	**N**	**[n (%)]**	**[n (%)]**	**[n (%)]**	**[n (%)]**	**[n (%)]**	**[n (%)]**

**Dementia in Alzheimer's disease**	**60**	20 (33.3)	22 (36.7)	9 (15.0)	7 (11.7)	7 (11.7)	7 (11.7)

**Vascular dementia**	**145**	15 (10.3)	12 (8.3)	13 (9.0)	4 (2.8)	4 (2.8)	8 (5.5)

**Dementia in Parkinson's disease**	**42**	13 (31.0)	11 (26.2)	3 (7.1)	28 (66.7)	28 (66.7)	3 (7.1)

**Unspecified dementia**	**330**	75 (22.7)	43 (13.0)	32 (9.7)	6 (1.8)	6 (1.8)	27 (8.2)

**Total**	**577**	**123 (20.2)**	**88 (15.3)**	**57 (9.9)**	**45 (7.8)**	**45 (7.8)**	**45 (7.8)**

^1 ^Double entries possible

There were significant differences in the proportion of patients treated with an antidepressant (GP: 17.6%; neurologist: 28.1%; *P *= 0.003) and benzodiazepine (GP: 11.4%; neurologist: 6.4%; *P *= 0.039) according to physician's specialisation. No difference was found for the treatment with antipsychotics (GP: 15.8%; neurologist: 14.3%; *P *= 0.635).

### Factors related to any anti-dementia drug therapy

Table 5 shows the adjusted odds ratio (AOR) for factors associated with the prescription of any anti-dementia drug. The AOR for receiving any anti-dementia drug was greater than 1 for the neurologists (AOR = 2.34; CI: 1.59-3.47), the diagnosis of Alzheimer's disease (AOR = 3.28; CI: 1.96-5.50), hypertension (AOR = 2.03; CI: 1.41-2.90), heart failure (AOR = 4.85; CI: 3.42-6.88), and neuroleptic therapy (AOR = 1.87; CI: 1.22-2.88). The odds for a prescription of any anti-dementia drug decreased with the diagnosis of vascular dementia (AOR = 0.64; CI: 0.43-0.95) and diabetes mellitus (AOR = 0.55; CI: 0.36-0.86). Patient age, sex, consultation type (first vs. follow up), multi-morbidity, and the year of prescription had no influence.

**Table 5:** Factors associated with any anti-dementia drug and *Ginkgo biloba*

Factor	Adjusted OR (95% CI)	
	**Any anti-dementia drug**	***Ginkgo biloba***

Sex		
Male	1	1
Female	1.040 (0.748-1.448)	0.406* (0.186-0.887)

Age [years]	0.988 (0.972-1.005)	1.058* (1.018-1.100)

Physician specialisation		
GP	1	1
Neurology	2.296* (1.557-3.384)	0.089* (0.034-0.232)

Type of dementia		
Unspecified dementia	1	1
Dementia in Alzheimer's disease	3.279* (1.959-5.496)	0.074* (0.035-0.156)
Vascular dementia	0.641* (0.433-0.948)	2.344 (0.701-7.837)
Dementia in Parkinson's disease	0.856 (0.491-1.493)	1.003 (0.315-3.196)

Multi-morbidity		
No	1	1
Yes	0.777 (0.497-1.214)	0.779 (0.403-1.504)

Hypertension	2.025* (1.413-2.901)	0.760 (0.334-1.732)

Heart failure	4.850* (3.419-6.880)	1.570 (0.684-3.603)

Chronic ischemic heart disease	0.822 (0.544-1.243)	0.751 (0.305-1.854)

Diabetes mellitus	0.553* (0.358-0.855)	2.549 (0.729-8.912)

Neuroleptic therapy	1.871* (1.217-2.876)	0.849 (0.393-1.833)

Antidepressant therapy	0.961 (0.649-1.422)	1.410 (0.655-3.038)

* AOR significantly different from 1

### Factors related to the prescription of *Ginkgo biloba*

The prescription of *Ginkgo biloba *was associated with sex, patient age, physician's specialisation, and the type of dementia (Table 5). Older patients (AOR = 1.06; CI: 1.02-1.10) were more likely to receive *Ginkgo biloba*. Female sex (AOR = 0.41; CI: 0.19-0.89), treatment by a neurologist (AOR = 0.09; CI: 0.03-0.23), and the diagnosis of Alzheimer's disease (AOR = 0.07; CI: 0.04-0.16) rapidly reduced the odds of being prescribed *Ginkgo biloba*, whereas co-morbidity, co-medication, consultation type, and the year of prescription had no influence.

## Discussion

In this paper we present the results of a secondary data analysis of electronic health record data from the EvaMed Network, a German network of CAM-oriented physicians. Overall, 25.6% of the patients with dementia were prescribed anti-dementia drugs.

The prescription of any anti-dementia drug was positively associated with physicians specialising in neurology, the diagnosis of Alzheimer's disease, neuroleptic therapy, co-morbidities hypertension, and heart failure. The odds for a prescription of any anti-dementia drug decreased with the diagnosis of vascular dementia and diabetes mellitus.

In coherence with their orientation, the phytopharmaceutical *Ginkgo biloba *was the most frequently prescribed anti-dementia drug overall (67.6% of all anti-dementia drugs) even though it has not been recommended in German dementia guidelines, while conventional anti-dementia drugs, such as cholinesterase inhibitors and memantine, ranged far behind. The prescription of *Ginkgo biloba *was positively associated with patient age, whereas the chance for the prescription of *Ginkgo biloba *was smaller than 1 for women, treatment by a neurologist, and the diagnosis of Alzheimer's disease.

### Study population

The median age of our study population was 81 years. Compared to the recently published study of Olsson et al. [32] conducted in nursing homes and special care units, our patient population was slightly younger due to the outpatient-character of this study but comparable with data from German health insurance provided by Koller et al. [33]. 69% of our patients were female, which is similar to the proportion of women in dementia patients in Germany (70% female) [34]. Considering the high age of our population the frequent number of co-morbidities is not surprising. Hypertension, congestive heart failure, and ischemic heart diseases are particularly common in this age and are well known as primers for vascular dementia and thus their occurrence is an indicator for dementia in our population (see Table 2).

### Anti-dementia drug therapy

In our study almost 26% of patients received anti-dementia drug therapy. According to the current ATC classification, *Ginkgo biloba *is listed in the group of anti-dementia drugs together with cholinesterase inhibitors and memantine. Thus we counted *Ginkgo biloba *as an anti-dementia drug although the literature is not consistent in this case. This has to be taken into consideration when comparing our results with other studies.

According to Formiga et al., 80.6% of the patients in a Spanish population received cholinesterase inhibitors or/and memantine for Alzheimer's disease [35]. In an Italian survey from 2007, Frisoni found a prescription rate of cholinesterase inhibitors in 90% of patients with Alzheimer's disease and 35-45% with vascular dementia. Other drugs, such as *Ginkgo biloba*, were prescribed less frequently, except for vascular dementia (20%) [36]. For Germany, Stoppe et al. found that only 24% of patients with Alzheimer's disease were prescribed cholinesterase inhibitors in 2004 [37], which is comparable to our study.

Although both the results of our study and those of Stoppe et al. [37] might be surprising at first, it has to be taken into account that especially the DEGAM guideline recommends a cautious and responsible handling of anti-dementia drugs in dementia patients and thus the results are in line with both the guidelines and the experience of the family physicians [25]. In Parkinson's disease, only one of 42 patients was mainly treated with rivastigmine which is recommended in the neurological guideline [19].

In 2006, a total of 50% of all prescribed anti-dementia drugs in Germany were cholinesterase inhibitors and memantine [13]. The most often prescribed anti-dementia drug in Germany was the cholinesterase inhibitor donepezil with 37% [38], which with a rate of 45% also plays a predominate role in our study. Traditional anti-dementia drugs like ergot alkaloids and piracetam, which are not recommended in the treatment guidelines, have declined in their prescribing rates over in recent years and, with only 6 patients, also play a minor role in our setting.

In our study only 8 out of 148 patients (5.4%) were prescribed both cholinesterase inhibitors and memantines during the study period. This is comparable to Hoffmann et al. who found only one anti-dementia drug in over 90% of his patients and Truter who only reported 5.3% of patients that were prescribed more than one active ingredient for Alzheimer's disease during one year (mostly donepezil or galantamine, and memantine) [38,39], whereas Formiga found combined therapy of cholinesterase inhibitors in 15.5% of patients [35]. While the DEGAM guideline disapproves combination therapies due to their low level of evidence, the DGN/DGPPN guideline refers to 2 RCTs and claims that an add-on therapy might be considered [18,19]. However, it has to be noted that in our study concomitant treatment with two drugs may also be a result of the course of therapy and might not reflect a factual combination therapy.

### Factors related to any anti-dementia drug therapy

According to Formiga, lower Barthel Index scores predict fewer prescriptions of pharmcological therapy in Alzheimer's disease [40]. Other factors like patient age, gender, co-morbidity, neuroleptic and antidepressant therapy, and hospital admission in the previous year show no significance after adjusting the multivariate logistic regression model.

We found that type of dementia, physician specialisation, co-morbidity, and neuroleptic therapy influenced the prescription of any anti-dementia drug (i.e. cholinesterase inhibitors, memantine and/or *Ginkgo biloba*). While odds ratios for Alzheimer's disease are presumably higher than those for vascular dementia, it is in accordance with the results of Formiga and Frisoni [36,40]. Moreover, there are several hints of different prescribing patterns of neurologists and GPs [25,38]. Ruof et al. found that neurologists prescribed cholinesterase inhibitors to 44.6% of their Alzheimer patients, while GPs only treated 9.0% of their patients with cholinesterase inhibitors [41]. Thus, physician specialisation seems to be a reasonable indicator for the prescription of any anti-dementia drug. A prescription of neuroleptics could indicate the presence of behavioural and psychological symptoms of dementia (BPSD) and these symptoms are more common in the middle or severe stages of dementia.

### Factors related to the prescription of *Ginkgo biloba*

The prescription of *Ginkgo biloba *was positively associated with patient age, whereas the chance for the prescription of *Ginkgo biloba *was smaller than 1 for women, patient treatment by a neurologist, and patient diagnosed with Alzheimer's disease. Co-morbidities and antidepressant or neuroleptic therapy did not reveal a significant impact, which might be due to the small sample size. Our results at first oppose the results of Nahin [42], who found that use of herbal and dietary supplements in older people in the United States was associated with female sex, a higher income, and a higher MMSE score in multivariate analysis. Although a subgroup analysis of factors related to the use of *Ginkgo biloba *had shown only race (white vs. nonwhite) as a significant factor, our results indicate that male patients are more likely to be prescribed *Ginkgo biloba*. At first this seems to counter studies which show that females are more likely to use CAM. However, it has to be noted that in dementia patients, the spouse might be the communication counterpart of the physician, which in the majority of cases of male dementia patients can be assumed to be the women. Thus, the predilection for CAM might be transferred to the partner.

Most of the physicians who asked for *Ginkgo biloba*, according to van den Bussche [25], assumed the potential effectiveness of *Ginkgo biloba*: 85% of the neurologists and 59% of the GPs attribute a moderate to high effectiveness. Thus it is not surprising that in our survey *Ginkgo biloba *was mainly prescribed by a neurologist. This is underpinned by the survey of Hillmer [43] on prescribing practices of Canadian family physicians with regards to Alzheimer's disease. The survey found that perception and self-reported knowledge are the most important factors that significantly predict lower prescribing rates. In the case of vascular dementia, there is no recommendation in the guidelines. Compared to other forms of dementia and taking the results of Hillmer into account, one therefore would assume a higher application of ginkgo preparations, which indeed was given in our data.

### Neuroleptics

The prescribing frequency of antipsychotic drugs in geriatric patients is commonly overestimated particularly with respect to the administration of benzodiazepines, which are not recommended (DEGAM, DGN/DGPPN) [44] due to higher mortality and a higher tendency to fall. Thus, both conventional and atypical neuroleptics should only be administered for a short period of time (DEGAM, DGN/DGPPN) [18,19]. According to Meyer et al., 28.4% of 2367 residents in 30 German nursing homes received neuroleptics and 23.6% were prescribed anxiolytic or hypnotic medication, mostly butyrophenone [45]. These rates do not differ from the 56.4% antipsychotic drugs being prescribed to 888 residents in Munich nursing homes [46]. Similar results were obtained from Mann et al. 2009 in Austria (antipsychotics 45.9%) [47], while the 31% reported in Italy was slightly lower [36]. Our results of only 14% thus are comparably low and might be explained by the CAM-orientation of the physicians. However, it is striking that benzodiazepines were mainly prescribed by GPs, while there was no significant difference between GPs and neurologists in the administration of neuroleptics.

### Concomitant use of anticholinergics and cholinesterase inhibitors

In recent years an intensive and still ongoing debate on the concomitant use of anticholinergics and cholinesterase inhibitors has emerged. It is assumed that anticholinergics may attenuate the effects of cholinesterase inhibitors. Other studies suggest that anticholinergics are prescribed to palliate side effects of cholinesterase inhibitors [48]. Data from registers from Australia and the USA reveal a proportion of joint prescriptions between 20 and 47% [49-51]. Our sample is too low to draw concrete conclusions about the concomitant use of anticholinergics and cholinesterase inhibitors; however, with only one of 40 patients being prescribed this combined treatment, one may assume that CAM-oriented physicians might be more cautions in the concomitant use of anticholinergics and cholinesterase inhibitors.

### Limitations

The present study has several important limitations which should be taken into account when interpreting the results. Firstly, additional data on the dementia diagnoses are lacking, such as a functional evaluation with the Barthel Index or an evaluation of the cognitive status with the Mini Mental State Examination (MMSE). The lack of objective tests for dementia massively increases the possibility of misclassification bias with respect to dementia type and severity. The proportion of Alzheimer's disease on all types of dementia normally should be between 50% and 70%. This is not reflected in our study which only found a proportion of about 10%. One explanation might be that GPs mainly (57%) classified the dementia as "unspecific". Secondly, although physician prescribing data were subjected to an internal review, coding inaccuracies cannot be ruled out entirely. Thirdly, data on subsequent medication use in patients who switched physicians were unavailable. Finally our study only presents data from a very small percentage of CAM physicians in Germany. The participation of 22 out of a possible 362 possible physicians does not support a broad generalisability of our findings nor does it allow carrying out detailed subgroup analyses. Thus, this analysis as such, should be viewed as a first insight into this field. In addition the present study also lacks a direct comparison group. Further research on this subject therefore would benefit from including a broader sample of CAM physicians as well as a stratified comparison group of conventional primary care physicians.

## Conclusions

The present study provides a systematic overview of everyday practice for treatment of dementia in primary care in physicians with a CAM focus. Both physicians and patients of our study are comparable to other studies, except for their CAM specification. We also found low prescribing rates of cholinesterase inhibitors and memantine, while *Ginkgo biloba *in contrast to the German guidelines was prescribed quite often. Comparing CAM-GPs and CAM neurologists, it is remarkable that GPs more often prescribe the not recommended butyrophenone, while neurologists mainly prescribe anti-dementia drugs. The administration of neuroleptics in both groups of physicians is comparable and considerably lower than in other studies. Further analysis regarding co-medication, co-morbidity, and the occurrence of critical combinations are of high interest to health services research.

## Competing interests

Dr. Vollmar was the first author of the DEGAM guideline for dementia. The other authors declare that they have no competing interests.

## Authors' contributions

EJ participated in the design of the study, acquisition of data, performed the statistical analysis, and drafted the manuscript. TO made substantial contributions to the interpretation of data and statistical analysis. HCV helped with the interpretation of the data and drafting and critical revision of the manuscript. MT and FS helped with the drafting and critical revision of the manuscript. HM conceived of the study and participated in its design and coordination. All authors read and approved the final manuscript.

## Pre-publication history

The pre-publication history for this paper can be accessed here:

http://www.biomedcentral.com/1471-2377/11/99/prepub
